# Full factorial design and dynamic modelling of silent and ultrasound-assisted lead and cadmium removal by porous biosorbent

**DOI:** 10.1038/s41598-022-10792-x

**Published:** 2022-04-28

**Authors:** S. Bdaiwi Ahmed, T. Dobre, F. Hashim Kamar, A. Mocanu, I. M. Deleanu

**Affiliations:** 1 Department of Chemical and Biochemical Engineering, University Politehnica of Bucharest, Polizu 1-7, 011061 Bucharest, Romania; 2 Environment and Water Directorate, Ministry of Science and Technology, Baghdad, Iraq; 3 Engineering Technical College, Middle Technical University, Baghdad, Iraq

**Keywords:** Chemical engineering, Environmental sciences

## Abstract

Present work aimed to analyse single and competitive lead and cadmium batch adsorption, using experimental studies and mathematical modelling. The experiments were conducted in silent and ultrasound-assisted systems, in aqueous environment, using grinded hazelnut shells as porous biosorbent. The influence of process factors (pH, adsorbent concentration, adsorbent particle size, and initial species concentration in liquid phase) on species removal efficiency was evaluated when process equilibrium was attained. The statistical study, following a 2^4^ factorial experimental design, allowed the development of a model to predict variables influence. Based on the obtained results a deeper analysis of the separation efficiency, depending on process factors, was conducted. The dynamic study was performed based on experimentally obtained removal rates, modelled considering species diffusion, with reversible kinetics of sorption inside solid particles. Hence, the dynamics of removal efficiency was determined for several representative experiments. The equilibrium isotherms data, best fitted by an appropriate Langmuir model, were used in the dynamic model to reduce the number of model parameters which normally require experimental identification.

## Introduction

Lead and cadmium, like almost all heavy metals, are a threat to terrestrial and aquatic ecosystems’ health. These metal ions and their complexes are known for their toxicity, non-degradability, bio-accumulation, bio-concentration and bio-magnification potential^1–3^. While the removal from water and wastewater has become mandatory, the process itself is not easy because of their high migration activity, solubility, and stability in aqueous environment^4^.

Numerous removal techniques were investigated so far, from classical phytoremediation, flocculation, or thermal treatment to cleaner biological processes or advanced membrane separation^5,6^. Among them, adsorption process (AP) is considered as one of the most feasible due to its energy-efficient and cost-effective operation, especially when large volumes of dilute solutions must be treated^7,8^. Furthermore, using improved and/or environmentally friendly (bio)sorbents, AP allows advanced pollutants removal without generating dangerous by-products^9^.

To achieve effective and economical adsorption, many influencing factors must be considered^10,11^, among which could be mentioned: a) co-ion presence and properties, that may cause synergic, antagonistic and/or non-interaction effects, b) adsorbent physicochemical characteristics (particle and pore size, area, surface chemistry) and properties (easy separation, good reusability, high adsorptivity), and c) medium conditions (pH, temperature, concentration, ionic strength, etc.). In the same time, as Di Natale et al., 2009 describes, the AP performance is the result of solute–solvent-sorbent interactions (thermodynamic basis) and diffusive-convective mass transfer of solute within porous sorbents (transport phenomena) at the same time^12^.

Considering the above mentioned, the attention given to AP modelling, as highly complex but economical and reliable procedure, has increased considerably in last years. Numerous studies have reported the development of mathematical models to predict the performance of AP. First models, known as surface complexation models, assumed that adsorption occurs due to acid–base reaction between ionized adsorbent surface sites and the ionic adsorbate^13^. These models, however proved to be useful only in the case of sorbents with homogeneous surface structure^12^. The usage of various (bio)sorbents, in a continuous search for pollutants sustainable removal, emphasised the complexity of solute-active site interactions. As a result the complexity of mathematical models permanently increased. Nowadays, both conventional models, derived from the classical isotherm equations, as well as the modern techniques like artificial neural networks are used for process design and optimization^2,8,11,12,14,15^.

In general, a sorption process model at sorbent particle level is successfully built when model parameters are identified based on batch phases contacting experiments. If so, the model can be used and useful in other operating approaches, like fixed bed adsorption for instance. Thus, we focused on the development of a dynamic mathematical model, validated by data fitting of both mono- and multi-solutes sorption experiments, which can be easily used to predict the performance of complex, non-competitive or competitive systems. Here, as in many cases of sorption dynamic modelling, the experimental data were used to calibrate the model and to determine some of its parameters.

HS was used for the experiments, as unconventional porous biosorbent characterized as economic since it’s in fact a waste that can be readily utilized, and its regeneration is not necessary. To overcome its relatively low adsorbing capacity, ultrasounds are used to enhance mass transfer into the pores and across the boundary layer as a result of high speed microjets, high-pressure shock waves and acoustic vortex microstreaming generation^16^. It is the first time, according to our knowledge, for an experimental and modelling study, to report effective diffusion coefficients and sorption rate constants for heavy metals removal by porous biosorbent in US-assisted adsorption.

## Material and methods

### Materials

As previously described by authors^17^, HS procured from Iraq local market, were thoroughly washed with distilled water, dried, grinded using a knife mill and sieved. In the experiments different sieved fractions of the grinded material were used, in accordance with the experimental plan (0.25–0.5 mm, 0.5–0.75 mm, 0.75–1 mm, 1.0–1.25 mm or 1.25–1.5 mm respectively). The average particle diameter was determined for each fraction through dynamic image analysis, using a Retsch (Microtrac MRB) Camsizer X2 particle size analyser, with measurement range of 0.8 μm–8 mm (X-fall dispersion), as recommended for particles with irregular shapes^18^.

All used reagents were analytical grade supplied by Merck KGaA, Germany: lead nitrate (Pb(NO_3_)_2_), and cadmium acetate dihydrate (Cd(CH_3_COO)_2_·2H_2_O). The pH of working solution was adjusted, when necessary, with 0.1 N NaOH or 0.1 N HCl and measured with a digital pH-meter.

### Methods and apparatus

#### Batch adsorption experiments

Two stock solutions were prepared: one of 1000 mg/L Pb(II), and one of 1000 mg/L Cd(II). Working solutions, of different concentrations, were obtained by dilution of stock solutions using distillate water.

The experiments were designed in triplicates, and the report is presenting the average values. Samples were taken at predetermined time intervals and filtered to remove the adsorbent. The residual metal concentration was determined using Agilent ICP Triple Quad (ICP-QQQ), equipped with an octopole collision-reaction cell positioned between two quadrupole mass filters and autosampler. ICP was used due to high throughput and enhanced sensitivity, suitable for trace element analysis. The samples were diluted using nitric acid 65% Suprapur supplied by Merck Millipore. The instrument was operated in single-quad mode.

To allow careful investigation of biosorption dynamics, the variation of the most important considered factors was performed in accordance with Table 1. The obtained results allowed us to conclude the optimal parameters for single and competitive systems. These parameters were used to study the influence of initial metal ion concentration (the equilibrium experiments).

**Table 1:** Experimental matrix for process factors in biosorption experiments.

Factor, m.u	Values	Operating mode	Fixed parameters in experiments
*d*_*p*_, mm	0.25 ÷ 1.50	US assisted	*pH* = 5.5, *m*_*HS*_ = 10 g/L, $\tau$ = 6 min
*pH*, -	3 ÷ 7	Silent adsorption	*d*_*p*_ = 0.75–1.00^.^mm, *m*_*HS*_ = 10 g/L, $n$ = 180 rpm, $\tau$ = 180 min
*m*_*HS*_, g/L	2.5 ÷ 15	Silent adsorption	*d*_*p*_ = 0.75–1.00^.^mm, *pH* = 5.5, $n$ = 180 rpm, $\tau$ = 180 min
		US assisted	*d*_*p*_ = 0.75–1.00^.^mm, *pH* = 5.5, $\tau$ = 6 min
*c*_*l0*_, mg/L	10 ÷ 100	Silent adsorption	*d*_*p*_ = 0.75–1.00^.^mm, *pH* = 5.5, $n$ = 180 rpm, $\tau$ = 180 min
		US assisted	*d*_*p*_ = 0.75–1.00^.^mm, *pH* = 5.5, $\tau$ = 6 min

Thus, silent batch experiments were conducted in 100 mL beakers containing 50 mL solution, of known concentration, under continuous stirring at 180 rpm, at 25 °C constant temperature, using a laboratory mechanical stirrer. According to Table 1 the solution pH, sorbent particle diameter, solid–liquid ratio and initial metal ion concentration were varied. Total stirring time was 180 min.

US-assisted batch experiments were conducted using a 750 Watt net power output and 20 kHz frequency Ultrasonic Processor Vibra Cell Sonics USA, model VCX750. The temperature was controlled and maintained by water circulating from a thermostatic bath. The amplitude for each experiment was set at 30%. The same parameters were considered: 50 mL solution of known concentration, and pH, 25 °C constant temperature. The contact time was significantly shorter, around 6 min. The above parameters were considered based on literature and previous practice^17,19^.

The sorption performance, in single and competitive systems, was expressed as removal efficiency ($R_{\tau }$), calculated according to the general Eq. (1):

1 $$ R_{\tau } = \left( {c_{l0} - c_{l\tau } } \right)/c_{l0} 100 $$

#### Statistical model for sorption efficiency

Considering operating parameters presented in Table 1, the influence of process factors on the maximum adsorption efficiency ($$R$$) was studied experimentally according to a 2^4^ full factorial design^20^, as will be further detailed in Table 2. The applied parameters and levels are as follows: adsorbent average particle diameter ($$x_{1} )$$, mm: 0.37, 0.87, 1.37; solution pH ($$x_{2} )$$: 3, 5, 7; solid liquid ratio ($$x_{3} )$$, g/L: 2.5, 8.5, 14.5; initial ion metal concentration in liquid phase ($$x_{4} )$$, mg/L: 10, 50, 90.

**Table 2:** Coded experimental design matrix as a function of the independent variables (x_1_, x_2_, x_3_ and x_4_) and separation efficiency (R) obtained for silent adsorption and US-assisted adsorption.

No	x_1_	x_2_	x_3_	x_4_	R, %							
					Silent sorption				US-assisted sorption			
					Single		Competitive		Single		Competitive	
					Pb(II)	Cd(II)	Pb(II)	Cd(II)	Pb(II)	Cd(II)	Pb(II)	Cd(II)
1	1	1	1	1	59.5	53.8	52.5	46.8	65.5	59.8	55.3	49.7
2	1	1	1	-1	87.2	79.1	77.2	68.1	90.2	84.1	69.3	63.1
3	1	1	− 1	1	71.9	63.2	55.9	48.2	75.9	67.5	59.9	51.7
4	1	1	− 1	− 1	69.3	64.9	58.3	52.5	77.6	70.2	61.6	54.3
5	1	− 1	1	1	56.7	49.7	59.3	53.6	59.7	52.9	44.3	38.5
6	1	− 1	1	− 1	61.4	56.7	49.4	41.7	63.5	58.3	48.5	43.1
7	1	− 1	− 1	1	51.3	48.8	37.1	30.5	53.3	49.8	53.7	47.7
8	1	− 1	− 1	− 1	61.1	57.3	55.3	53.4	66.2	59.7	51.9	43.7
9	− 1	1	1	1	75.3	70.9	49.3	47.9	83.5	74.6	67.8	59.7
10	− 1	1	1	− 1	89.6	84.9	73.3	63.2	93.6	86.9	78.2	70.7
11	− 1	1	− 1	1	67.2	59.5	49.9	40.8	75.5	68.8	60.9	53.3
12	− 1	1	− 1	− 1	80.1	78.8	65.1	63.8	83.1	80.3	79.9	65.5
13	− 1	− 1	1	1	64.5	57.7	49.9	41.8	74.7	67.9	59.5	52.2
14	− 1	− 1	1	− 1	76.1	67.9	58.9	52.3	76.9	68.6	63.7	52.9
15	− 1	− 1	− 1	1	49.8	44.7	36.7	31.8	55.5	49.9	41.9	34.1
16	− 1	− 1	− 1	− 1	55.5	50.7	42.7	37.7	62.7	56.2	48.3	41.8
17	0	0	0	0	74.8	70.1	57.8	44.5	94.2	87.5	73.3	59.5
18	1.414	0	0	0	71.1	66.8	51.1	42.4	81.1	76.8	64.1	61.9
19	− 1.414	0	0	0	79.3	74.7	59.2	47.7	88.7	85.8	71.7	60.7
20	0	1.414	0	0	44.7	38.7	52.7	44.7	54.5	48.7	39.5	33.8
21	0	− 1.414	0	0	28,2	22.5	31.1	19.8	39.6	31.4	27.5	17.4
22	0	0	1.414	0	76.8	72.1	59.8	45.6	87.8	82.9	71.8	66.9
23	0	0	− 1.414	0	52.7	48.8	38.3	31.9	63.9	59.8	49.9	42.8
24	0	0	0	1.414	55.6	49.3	37.6	30.3	57.6	50.9	42.6	36.7
25	0	0	0	− 1.414	79.5	74.3	75.1	68.8	90.5	84.3	73.5	68.4

The same experimental procedures were applied. Silent batch experiments were conducted in 100 mL beakers containing 50 mL solution, under continuous stirring at 180 rpm, for 180 min, at 25 °C constant temperature, using a laboratory mechanical stirrer. US-assisted batch experiments were conducted under controlled temperature, using 50 mL solution. The amplitude for each experiment was set at 30%. According to the experimental plan the solution pH, sorbent particle diameter, solid–liquid ratio and initial metal ion concentration were varied. Experimental data were fitted to determine the coefficients of the regression model using the following general Eq. ^20^:

2 $$  \begin{aligned}   R_{n}^{m}  =  & \beta _{{0n}}^{m}  + \mathop \sum \limits_{{i = 1}}^{4} \beta _{{in~}}^{m} x_{{in}}^{m}  + \mathop \sum \limits_{{j = 2,j \le 4}}^{4} \beta _{{1j~n}}^{{m~}} ~x_{{1n}}^{m} x_{{jn}}^{m}  + \mathop \sum \limits_{{j = 3,j \le 4}}^{4} \beta _{{2j~n}}^{{m~}} ~x_{{2n}}^{m} x_{{jn}}^{m}  + \mathop \sum \limits_{{j = 4}}^{4} \beta _{{3j~n}}^{{m~}} ~x_{{3n}}^{m} x_{{jn}}^{m}  + \beta _{{14n}}^{m} x_{{1n}}^{m} x_{{4n}}^{m}  \\     &  + \mathop \sum \limits_{{j = 2.k = j + 1}}^{4} \beta _{{1ijkn}}^{m} x_{{in}}^{m} x_{{jn}}^{m} x_{{kn}}^{m}  + \beta _{{234n}}^{m} x_{{2n}}^{m} x_{{3n}}^{m} x_{{4n}}^{m}  + \beta _{{1234n}}^{m} x_{{1n}}^{m} x_{{2n}}^{m} x_{{3n}}^{m} x_{{4n}}^{m}  + \mathop \sum \limits_{{i = 1}}^{4} \beta _{{iin}}^{m} \left( {x_{i}^{2}  - x_{{ci}}^{2} } \right) \\  \end{aligned}   $$

#### Equilibrium experiments

Adsorption isotherms experiments were carried out considering optimal conditions of previous experimental study: pH = 5.5, and adsorbent dose 10 g/L. For the experiments, the adsorbent with particle diameter between 0.75 and 1.0 mm was used, and temperature was maintained at 25 °C.

Initial metal ion/ions solutions concentration ranged between 10 mg/L and 100 mg/L. The experiments were performed in silent mode and US-assisted. Experimentation followed the same procedures as detailed before (See Sect. “Statistical model for sorption efficiency”).

The amount of Pb(II) and Cd(II) adsorbed per unit mass of biosorbent at equilibrium was calculated using the following equation:

3 $$ c_{se} = \left( {c_{l0} - c_{le} } \right) \cdot V_{l} /{\text{m}}_{s} $$

The obtained results concerning adsorption isotherms for single species, were mathematically described using a Langmuir type model, which in linear expression can be described as:

4 $$ \frac{1}{{c_{se} }} = \frac{a}{{c_{le} }} + \frac{1}{Q} $$

#### Sorption dynamics experiments

Experiments were performed for single and competitive adsorption, in silent and US-assisted approach. Working temperature was 25 °C, the pH was adjusted to 5.5, average adsorbent particle diameter was 0.87 mm, the stirring rate was fixed at 180 rpm (for silent adsorption) and solid concentration in the system was 10 g/L in all cases. Silent batch experiments were conducted in 100 mL beakers containing 50 mL solution, under continuous stirring for 180 min. US-assisted batch experiments were conducted under controlled temperature, using 50 mL solution. The amplitude for each experiment was set at 30%.

The concentration of transferable species in the liquid phase was measured at specific time intervals.

## Modelling of experimental sorption dynamic

Modelling of batch sorption dynamics for one or more components it’s a challenging issue due to number of parameters, equations, and restrictions describing the process^20,21^. Taking into account our expertise and literature survey^20–25^, we consider that the sorption rate inside HS particle is controlled by species diffusion and their reaction with the sorbent active sites. To establish the correspondent mathematical model the following assumptions can be considered: i) perfect mixing of liquid phase; ii) HS/sorbent particles are porous, spherical with the equivalent radius, $R_{p}$ and are surrounded by a perfectly mixed liquid; iii) depending on experiment type the active species diffuse within solid particle pores, where these are adsorbed onto active surface sites; iv) overall adsorption rate of species depends on the competition between adsorption and desorption processes; v) kinetics of sorption process is linear with respect to coverage degree of surface pores and species concentration in liquid phase; vi) kinetics of desorption process is linear with respect to species concentration in solid phase; vii) sorption experiments follow the isothermal process conditions.

According to the above mentioned, the following system of equations and restrictions will represent the mathematical model for the dynamics of simultaneous Pb(II) and Cd(II) sorption on HS:

- Conservation of species of $c_{i}$ concentration in the liquid within porous sorbent particle:
  5 $$ \frac{{\partial c_{i} }}{\partial \tau } = D_{efi} \left( {\frac{{\partial^{2} c_{i} }}{{\partial r^{2} }} + \frac{2}{r}\frac{{\partial c_{i} }}{\partial r}} \right) - v_{Ri} \left( {c_{i} ,c_{si} } \right) $$
- Kinetic equation of species for to the solid phase (HS particle):
  6 $$ \frac{{\rho_{p} }}{{ \varepsilon_{p} }}\frac{{\partial c_{si} }}{\partial \tau } = v_{Ri} \left( {c_{i} ,c_{si} } \right) $$
- Overall adsorption rate equation of species:
  7 $$ v_{Ri} \left( {c_{i} ,c_{si} } \right) = k_{ai} \left( {1 - \frac{{\mathop \sum \nolimits_{i = 1}^{N} \alpha_{i} c_{si} }}{Q}} \right)c_{i} - k_{di} \frac{{\rho_{p} }}{{\varepsilon_{p} }}c_{si} $$
- Balance of species transfer rate with respect to solid phase (HS particle):
  8 $$ - V_{l} \frac{{dc_{li} }}{d\tau } = m_{s} \frac{{dc_{si mn} }}{d\tau } $$
- Equation for momentary mean concentration of species *i* in solid phase:
  9 $$ c_{si \,mn} \left( \tau \right) = \frac{1}{{R_{p} }}\mathop \smallint \limits_{0}^{{R_{p} }} c_{si} \left( {r,\tau } \right)dr $$
- Initial conditions for species concentration in solid and liquid field:
  10 $$ \begin{aligned} & \tau = 0, \;\;\;0 \le r < R_{p} ,\;\;\;c_{i} = 0,\;\,\;c_{si} = 0 \\ & \tau = 0, \;\;\; r > R_{p} , \;\;\; c_{li} = c_{li0} \\ \end{aligned} $$
- Boundary conditions for species diffusion (Eq. (5)) in solid HS particle:
  11 $$ \begin{aligned} & \tau > 0, \;\;\;r = 0, \;\;\;\frac{{{\text{dc}}_{i} }}{{{\text{dr}}}} = 0,\;\;\;\frac{{{\text{d}}c_{si} }}{{{\text{dr}}}} = 0 \\ & \tau > 0,\;\;\; r = R_{p} ,\;\;\;V_{l} \frac{{dc_{li} }}{d\tau } = - D_{efi} S\left( {\frac{{dc_{i} }}{d\tau }} \right) \\ \end{aligned} $$

The system of equations and restrictions (5 – 11) was numerically transposed in a parameters identification problem using an adequate finite differences method. Thus, values of momentary mean concentration of species in liquid phase, depending on known parameters ($R_{p}$, $\varepsilon_{p}$, $\rho_{p}$, $\alpha_{i}$, $Q$, $c_{i}$_*,*_$c_{li0}$), and unknown parameters ($D_{efi}$, $k_{ai}$, $k_{di}$), are correlated to the measured mean concentration of species in liquid phase. The values of unknown parameters were identified by minimizing the objective function described by Eq. (12):

12 $$ f\left( {D_{efi} ,k_{ai} ,k_{di} } \right) = \mathop \sum \limits_{j} \left[ {c_{li \, mn} \left( {\tau_{j} } \right) - c_{liexp \,mn} \left( {\tau_{j} } \right)} \right]^{2} $$

It is important to mention that using the equilibrium data we can obtain an estimation for reaction constants ratio for both sorption and desorption processes. Also, from here, the total sorption capacity of the HS sorbent can be obtained. If for Eq. (7) we consider the equilibrium condition and single specie sorption, the result is previous Eq. (4) where:

13 $$ a = \left( {\frac{{k_{d} }}{{k_{a} }}} \right)\left( {\frac{{\rho_{p} }}{{\varepsilon_{p} }}} \right) $$

For competitive sorption on HS, when equilibrium conditions are applied to Eq. (7) the system of Eqs. (14) and (15) can be obtained, which will be used to determine the values for $Q$, $\alpha_{1}$, $\alpha_{2}$, $k_{d1} /k_{a1}$ and respectively $k_{d2} /k_{a2}$:

14 $$ \frac{{k_{d1} }}{{k_{a1} }}\frac{{\rho_{p} }}{{\varepsilon_{p} }} = \left( {1 - \frac{{\alpha_{1} c_{s1e} + \alpha_{2} c_{s2e} }}{Q}} \right)\left( {\frac{{c_{l1e} }}{{c_{s1e} }}} \right) $$

15 $$ \frac{{k_{d2} }}{{k_{a2} }}\frac{{\rho_{p} }}{{\varepsilon_{p} }} = \left( {1 - \frac{{\alpha_{1} c_{s1e} + \alpha_{2} c_{s2e} }}{Q}} \right)\left( {\frac{{c_{l2e} }}{{c_{s2e} }}} \right) $$

## Results and discussion

Native and US-treated HS, as lignocellulosic material suitable to be used as biosorbent, was previously investigated and characterised^17^. Briefly, Scanning Electron Microscopy revealed that US determines mechanical ruptures with formation of pores and smother surfaces, while X-ray diffraction showed no modification on cellulose crystalline structure under US treatment. Fourier Transform Infrared spectra emphasised the complex material composition, with many functional groups interacting with metal ions. Thus, the sorption on this vegetal structure was proved as the result of a combination of mechanisms: physical adsorption, ion exchange, electrostatic interactions and formation of complexes^17,19,26,27^. Furthermore, ultrasounds significantly intensify this process due to acoustic cavitations’ development inside and outside of sorbent particle^28–30^.

### Statistical study of sorption efficiency

The influence of the main studied parameters on separation efficiency of Pb(II) and Cd(II) in single and competitive, silent and US assisted adsorption was analysed. Coded experimental design matrices and experimentally obtained sorption efficiencies, are presented in Table 2.

In all cases, for the significance of each coefficient, the reproducibility was obtained from four replicated experiments in the centre of experimental plan (see line 17, Table 2).

The equation of the statistical model, which is in fact a polynomial regression, it’s a consequence of the experimental investigation plan. In this specific case, when the experimental design followed a second order orthogonal plane, this equation has the general form given by the relation (2). Based on this equation, the obtained particularizations are described by the following Eqs. (16 – 23):

16 $$ R_{1}^{0} = 65.57 + 6.19x_{2} - 4.21x_{4} + 3.24\left( {x_{1}^{2} - 0.8} \right) - 11.13\left( {x_{2}^{2} - 0.8} \right) + 4.41\left( {x_{4}^{2} - 0.8} \right) $$

17 $$ R_{2}^{0} = 60.26 + 6.08x_{2} - 4.61x_{4} + 3.312\left( {x_{1}^{2} - 0.8} \right) - 11.74\left( {x_{2}^{2} - 0.8} \right) + 4.41\left( {x_{4}^{2} - 0.8} \right) $$

18 $$ R_{3}^{0} = 53.05 + 5.06x_{2} + 3.03x_{3} - 4.92x_{4} - 2.3x_{2} x_{4} - 2.99x_{2} x_{3} x_{4} + 2.77\left( {x_{1}^{2} - 0.8} \right) - 3.63\left( {x_{2}^{2} - 0.8} \right) $$

19 $$ \begin{aligned} R_{4}^{0} = & 45.65 + 5.07x_{2} + 2.25x_{3} - 4.94x_{4} - 1.8x_{2} x_{4} - 3.39x_{1} x_{2} x_{3} x_{4} + 4.01\left( {x_{1}^{2} - 0.8} \right) - 2.29\left( {x_{2}^{2} - 0.8} \right) + \\ & \;6.4\left( {x_{4}^{2} - 0.8} \right) \\ \end{aligned} $$

20 $$ R_{1}^{1} = 75.51 + 6.53x_{2} + 5.58\left( {x_{1}^{2} - 0.8} \right) - 13.43\left( {x_{2}^{2} - 0.8} \right) $$

21 $$ R_{2}^{1} = 66.54 + 6.69x_{2} - 3.67x_{4} + 6.26\left( {x_{1}^{2} - 0.8} \right) - 14.51\left( {x_{2}^{2} - 0.8} \right) $$

22 $$ R_{3}^{1} = 58.22 + 5.98x_{2} - 2.99x_{1} x_{3} + 6.16\left( {x_{1}^{2} - 0.8} \right) - 11.12\left( {x_{2}^{2} - 0.8} \right) $$

23 $$ R_{4}^{1} = 50.77 + 5.74x_{2} - 2.71x_{1} x_{3} + 6.54\left( {x_{1}^{2} - 0.8} \right) - 11.16\left( {x_{2}^{2} - 0.8} \right) + 3.46\left( {x_{3}^{2} - 0.8} \right) $$

The results of the statistical model described by Eq. (16 – 23) show, quantitatively, that US increases the separation efficiency with up to 10 percent (comparison between corresponding free term values). Also, comparing the equations describing single Pb(II) and Cd(II) sorption with the equations obtained for competitive sorption, one can see that the terms expressing the factors interaction are missing in the first cases, while in the competitive sorption they are significant, especially for silent experiments.

Thus, for a given biosorbent structure (porous HS particles in our case), the equations clearly indicate that process equilibrium, and consequently the maximum effectiveness of the separation, is mainly influenced by 3 factors in the following order: $pH$ > $c_{l0}$ > $m_{HS}$, with an average variability, depending on specific case.For instance, the variability of pH is ranging from − 4.5% to + 11.3%. However, in all above equations a quadratic dependence can be noticed $\left( {x_{1}^{2} - 0.8} \right)$. Its contribution is between − 2.45% and + 0.65% for silent sorption, and between − 5.2% and + 1.7% in the case of US-assisted sorption. This could be explained, as described before, by mechanical structure modification under US field.

**Figure 1:**
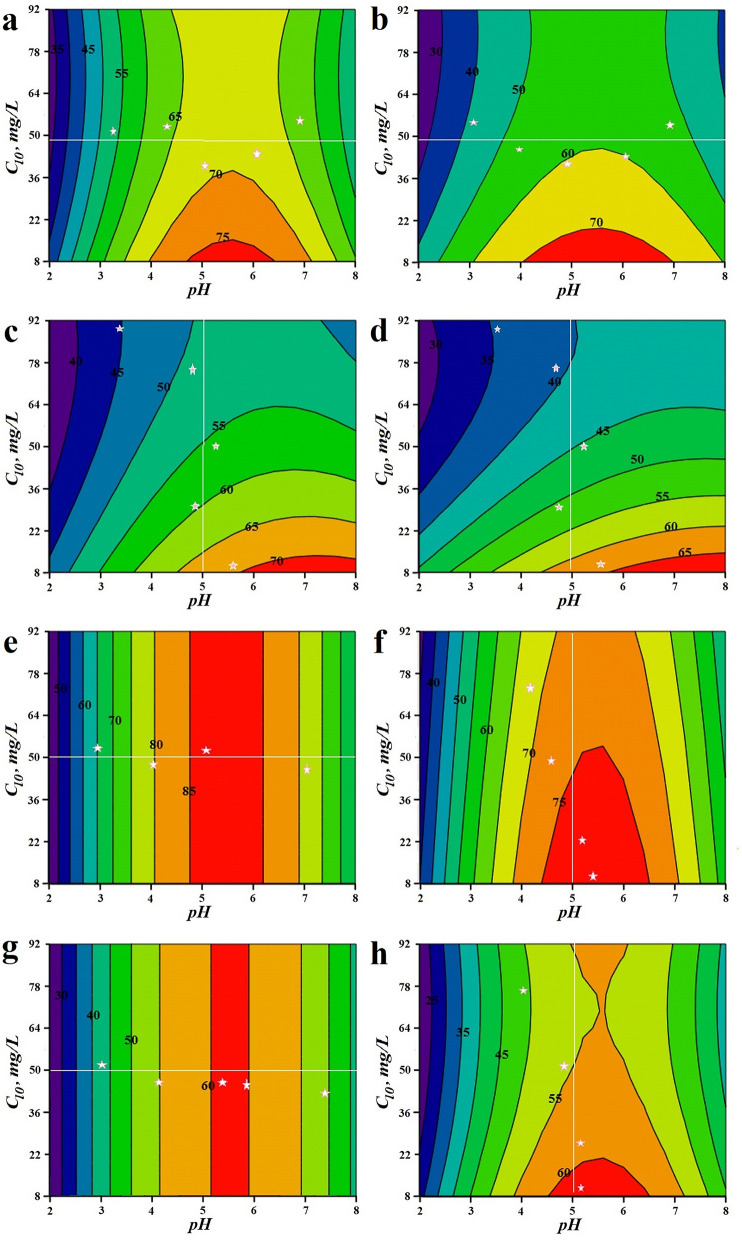
Influence of pH and initial Pb(II) concentration (**a**,** c**,** e**,** g**) / initial Cd(II) concentration (**b**,** d**,** f**,** h**) on separation efficiency: (**a**,** b**) single silent sorption, (**c**,** d**) competitive silent sorption, (**e**,** f**) single US-assisted sorption, (**g**,** h**) competitive US-assisted sorption. Experimental points (white stars) were obtained for $d_{p}$ = 0.87 mm, $m_{HS}$ = 8 g/l, pH from 3 to 7 (**a**,** b**,** e**,** g**), and $c_{l0}$ from 10 to 90 mg/l (**c**,** d**,** f**,** h**).

The effect of pH and initial concentration of transferable species (metal ion/s) on separation efficiency is presented in Fig. 1. Here the other two factors, namely sorbent particle size and sorbent dosage were maintained in the centre of the experimental plane. In addition to the statistical model representation of $pH$ and $c_{l0}$ influence on separation efficiency, in Fig. 1 experimental data are added. The experimental data (represented as white stars) were also obtained for pH and the initial ion metal concentration as manipulated variables, while the other variables were maintained in the centre of the experimental plane. The white line in Fig. 1 allows us to compare theoretical data (resulted from statistical model) with the experimental obtained ones.

As example, with reference to the Fig. 1a, for an initial metal ion concentration of 50 mg/L, and pH = 3, Pb(II) theoretical (from model) and experimental separation efficiency is 51% and 53% respectively. Overall, we can notice a satisfactory agreement between experimental and model data. An important finding however refers to US-assisted single and competitive adsorption, where, as can be seen in Fig. 1e, and Fig. 1g, the separation efficiency depends only on solution *pH*.

Concerning adsorbent particle size and solid liquid ratio, the two parameters could be connected to specific area of porous particles^31^. Thus, the internal area, directly related to the total weight of used adsorbent (here HS) determines metal removal, while the external area becomes important only for very large particles. This limited contribution of external surface area was in fact generalized for natural biomaterials, as mentioned in previous studies, even in the absence of ultrasounds^32^. While, clearly, the highest removal efficiency of Pb(II) and Cd(II) was obtained for single sorption experiments, the above observation is applicable for competitive adsorption experiments also. At the same time, the high percent removal obtained in a very short time, gives a good indication that ultrasounds penetrate the pores, enhancing convective mass transport due to acoustic cavitations^33,34^. Our findings confirm that US in solid–liquid systems increase sorption capacity and especially the sorption rate due to the effect of cavitation^35^. The cavitation bubbles collapsing in the liquid phase generate shock waves and shear forces and consequently advanced solids dispersion^36,37^.

### Modelling of experimental sorption dynamic

Another direction of present research refers to identification of a quantitative expression characterizing the interphases equilibrium for Pb(II) and Cd(II) sorption, in single and competitive systems, using grinded HS particles. The main objective was to determine biosorption process equilibrium constant, expressed as ratio of desorption and sorption rate constants. As previously shown, this rate constants ratio (equilibrium constant) is important in identifying the characteristic parameters of sorption dynamic model at particle level. It must be said that all the above presented data, concentrated in eq. system (16 – 23), are in fact an indirect expression of the interphase sorption equilibrium. Using these equations, the interphase equilibrium can be expressed as Eq. (4) or as the Eqs. (14, 15) or as equivalents, depending on the characteristics of the adsorption system.

The results of the sorption dynamic evaluation presented in Table 3, were obtained for pH 5.5, considered as optimum value. Specifically, equilibrium ion metal concentration in the liquid phase, and the equilibrium/maximum sorption efficiency, were determined experimentally while the equilibrium ion metal concentration in the solid phase, was determined using Eq. (3). All fixed and manipulated parameters are described in Table 3.

**Table 3:** Experimental parameters and equilibrium data for Pb(II) and Cd(II) sorption on HS at 25 °C.

Fixed factors, m.u	Manipulated factors, m.u			$c_{l0}$, mg/L	10	25	50	75	100
*d*_*p*_ = 0.87 mm *m*_*HS*_ = 10 g/L *pH* = 5.5	Silent sorption	Single system	Pb(II)	$c_{le}$, mg/L	0.96	4.42	12.15	40.05	60.71
				$R$, %	90.4	82.3	75.7	46.6	39.3
				$c_{se}$, g/g	0.904	2.058	3.785	3.495	3.931
			Cd(II)	$c_{le}$, mg/L	1.14	6.36	14.95	47.85	72.5
				$R$, %	87.6	74.5	70.1	36.2	27.5
				$c_{se}$, g/g	0.886	1.865	3.505	2.715	2.75
		Competitive system	Pb(II)	$c_{le}$, mg/L	0.73	3.21	10.82	21.77	33.11
				$R$, %	84.4	73.9	56.7	44.6	33.8
				$c_{se}$, g/g	0.427	0.929	1.418	1.672	1.69
			Cd(II)	$c_{le}$, mg/L	1.57	5.32	13.97	26.17	38.25
				$R$, %	68.6	57.7	44.1	30.2	23.5
				$c_{se}$, g/g	0.313	0.721	1.106	1.131	1.171
	US-assisted sorption	Single system	Pb(II)	$c_{le}$, mg/L	0.26	0.69	6.65	31.82	54.7
				$R$, %	98.4	97.1	86.7	57.6	45.3
				$c_{se}$, g/g	0.984	2.435	4.335	4.329	4.53
			Cd(II)	$c_{le}$, mg/L	1.04	2.85	11.45	33.61	62.5
				$R$, %	89.6	87.6	77.1	53.2	37.5
				$c_{se}$, g/g	0.896	2.215	3.855	4.145	3.751
		Competitive system	Pb(II)	$c_{le}$, mg/L	1.08	3.09	7.83	20.41	32.63
				$R$, %	78.4	75.3	68.6	45.6	34.8
				$c_{se}$, g/g	0.392	0.941	1.718	1.715	1.739
			Cd(II)	$c_{le}$, mg/L	1.77	4.93	11.72	25.42	39.25
				$R$, %	64.6	60.5	50.1	33.2	24.5
				$c_{se}$, g/g	0.323	0.758	1.208	1.218	1.225

**Figure 2:**
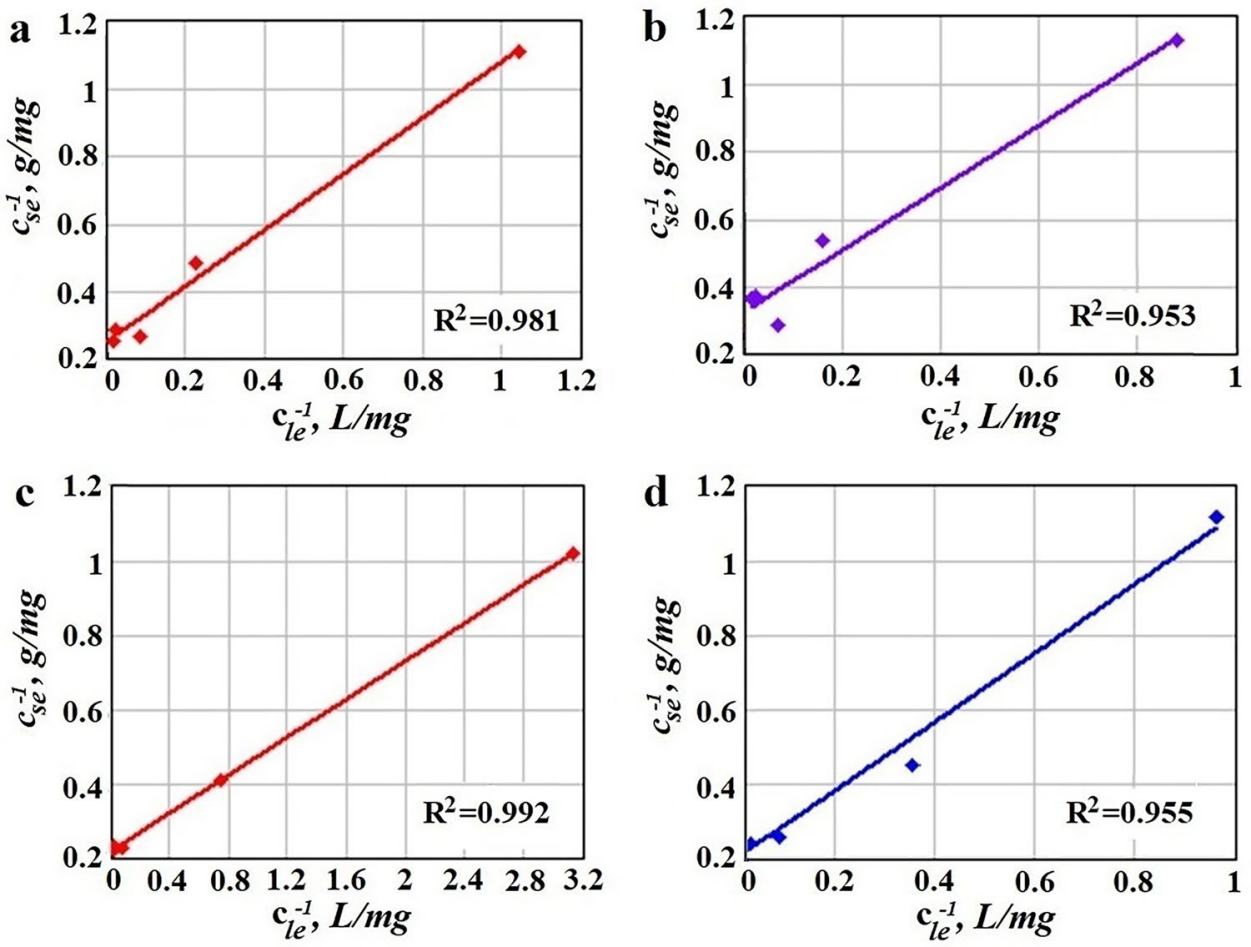
Linear Langmuir isotherm plots (t = 25 °C) for: single silent adsorption (**a**) Pb(II), (**b**) Cd(II) and single US-assisted adsorption (**c**) Pb(II), (**d**) Cd(II); points represent experimental data.

Using the parameters presented in Table 3, Langmuir isotherms for single silent and US-assisted sorption of Pb(II) and Cd(II) were plotted (Fig. 2).

Langmuir adsorption isotherm model assumes monolayer adsorption on homogenous surfaces and no interaction between adsorbed species^38^. Most of reported studies on heavy metals biosorption reported this model as one of the best in fitting the experimental results^6^. As can be seen in Fig. 2, Langmuir model is suitable to describe monocomponent sorption in this study also, with values of the correlation coefficient R^2^ greater than 0.95.

The sorption characterizing parameters are presented in Table 4. The results, as expected, confirm the difference between silent and US-assisted sorption performance. Thus, higher values for adsorbed amount at equilibrium were obtained in US-assisted experiments. Comparing the results of Table 4, it should also be noted that values of total sorption capacity obtained for competitive systems exceeds any of the individual sorption capacities, whether ultrasonic field is applied or not, so it can be assumed that Pb(II) and Cd(II) are not oriented towards the same sorption sites. Also, higher values of equilibrium constants , correlated with lower sorption capacities obtained for Cd(II) could be explained by its lower tendency to form hydrolysis products^39,40^. Similar findings regarding factors influence on separation efficiency and solid–liquid equilibrium for Pb(II) and Cd(II) adsorption in single and competitive systems could be found in many other published reports^26,41,42^.

**Table 4:** Sorption isotherm parameters (${\text{Q}}$, ${\upalpha }_{1}$, ${\upalpha }_{2}$, ${\text{K}}_{{\text{e}}}$) and dynamic model parameters (${\text{D}}_{{{\text{ef}}}}$ and ${\text{k}}_{{\text{a}}}$) at 25 °C, *pH* = 5.5 and ${\text{m}}_{{{\text{HS}}}}$ = *10 g/l.*

Langmuir constants, m.u	Silent adsorption				US-assisted adsorption			
	Single		Competitive		Single		Competitive	
	Pb(II)	Cd(II)	Pb(II)	Cd(II)	Pb(II)	Cd(II)	Pb(II)	Cd(II)
Q, mg/g	4.096	3.051	5.013		4.555	3.626	5.492	
α_1_, α_2_, -	-	-	α_1_ = α_2_ = 0.5		−	-	α_1_ = α_2_ = 0.5	
*K*_*e*_, -	5.95 10^–4^	6.58 10^–4^	6.02 10^–3^	8.11 10^–3^	8.94 10^–4^	7.62 10^–4^	5.51 10^–3^	7.61 10^–3^
$k_{a}$, s^-1^	2.91 10^–4^	4.35 10^–4^	9.05 10^–5^	4.13 10^–5^	4.05 10^–3^	6.43 10^–3^	3.01 10^–4^	2.25 10^–4^
$D_{ef}$, cm^2^/s	2.9 10^–8^	3.5 10^–8^	1.5 10^–8^	1.6 10^–8^	6.4 10^–7^	6.1 10^–7^	6.3 10^–7^	6.1 10^–7^

**Figure 3:**
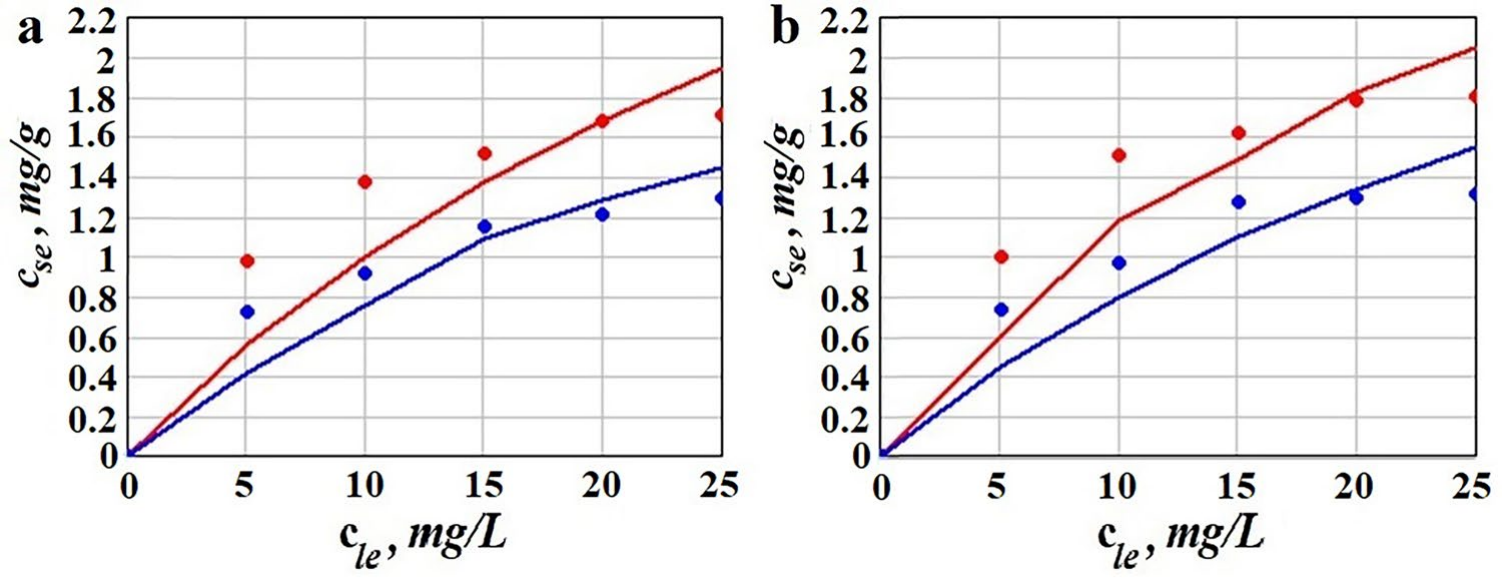
Isotherm plots for competitive Pb(II) and Cd(II): (**a**) silent, (**b**) US-assisted sorption; continuous line obtained using Eqs. (13,14), points represent experimental data at 25 °C; red colour is for Pb(II), blue for Cd(II).

The isotherm plots for competitive sorption presented in Fig. 3 were obtained using the parameters identified from Eqs. (14, 15), as described before. They show the differences between sorption equilibrium in silent and US-assisted approach; specifically, for $c_{le} > 5$ mg/L, $c_{se}$ is 8–10% higher in US-assisted adsorption. For simultaneous removal of Pb(II) and Cd(II) the competition between species for the sorption sites was observed. This could be explain by low specific surface of the biosorbent. As can be seen in Table 4 the adsorbed amount at equilibrium is quite poor comparing with advanced modified sorbent nanostructures^43^.

### Identification of parameters characterizing biosorption dynamics

Two parameters are considered to characterize biosorption dynamics: the effective diffusion coefficient and the adsorption rate constant. Generally, in the theory of Fickian diffusion, the diffusion coefficient is a constant (usually measured in cm^2^ s^−1^), that can only be determined experimentally, expressing the material properties^44^. In the process of adsorption, the effective diffusion coefficient is the quantitative parameter that considers porous adsorbent properties and the properties of the transferred component at the same time, in specific adsorption conditions^45,46^. To determine their values, experimentally obtained data, according to procedure detailed in subchapter 2.2.4, and the dynamic model Eqs. (5–11) were used.

In the case of single sorption systems, the identification algorithm consisted in: i) numerical transposition of dynamic model equations as a function showing species dynamic concentration in the liquid , ii) building the objective function (Eq. 12) for parameters identification based on experimental values of the concentration, iii) minimization of the objective function.

A similar procedure was used for competitive sorption. In this case however, instead of a numeric function with two parameters, $c_{l} \left( {D_{ef} , k_{a} } \right)$, an equivalent function with four parameters, $c_{l} \left( {D_{ef1} , k_{a1} , D_{ef2} , k_{a2} } \right)$ was used. Since it’s not recommended to identify more than two parameters based on a single data set^20^, it was considered that the values of $D_{ef1}$, $D_{ef2}$, $k_{a1}$, and $k_{a2}$ are close to those characterizing single component sorption.

**Figure 4:**
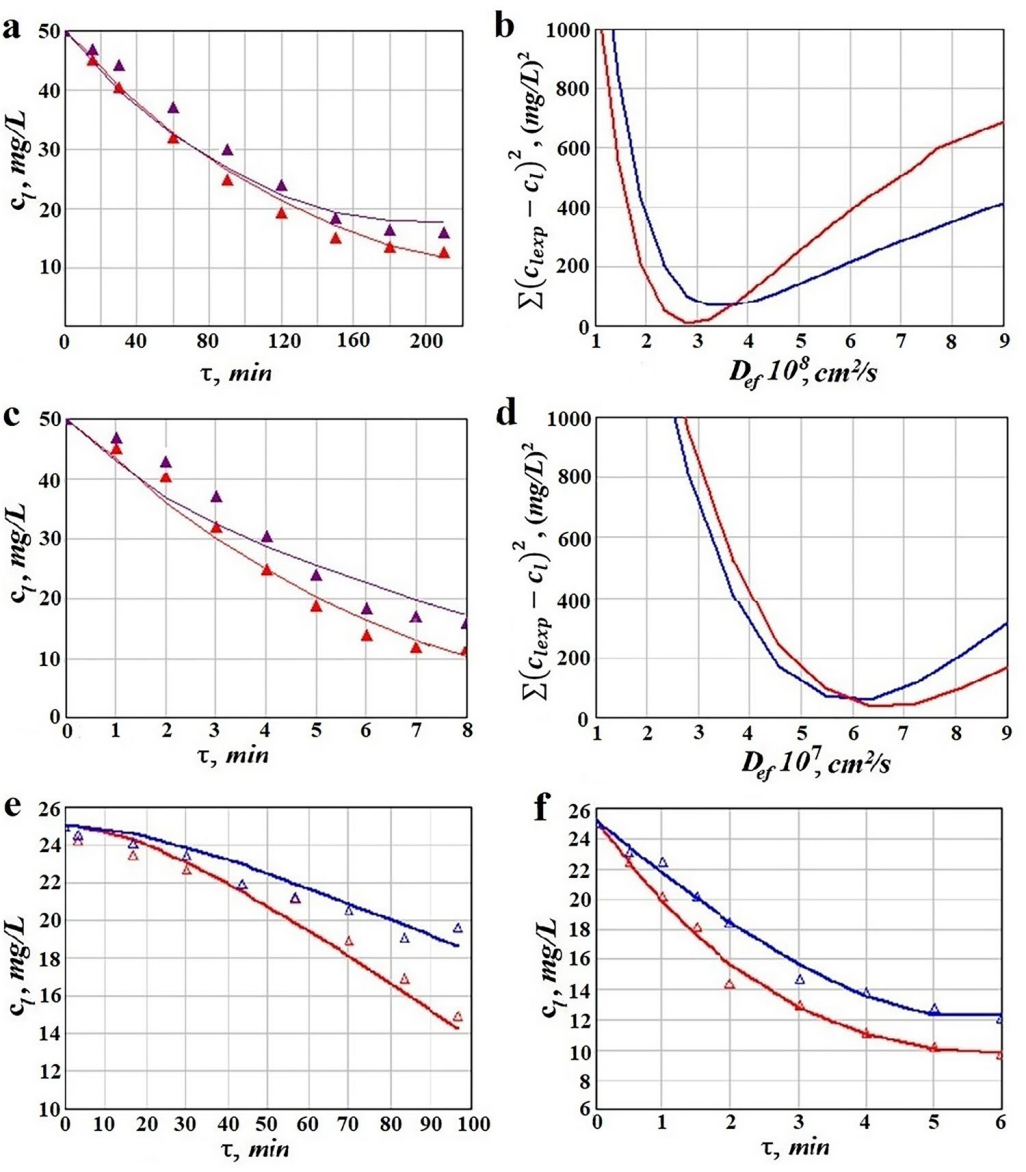
Dynamics of Pb(II) and Cd(II) concentration in liquid phase in time for: (**a**) single silent adsorption, (**b**) single US-assisted adsorption, (**e**) competitive silent adsorption, (**f**) competitive US-assisted adsorption, and sensitivity of mean square deviation of concentrations with respect to the diffusion coefficient for: (**b**) single silent adsorption, and (**d**) single US-assisted adsorption; continuous lines represents model data based on parameters given in Table 3, points represent experimental data; red colour is for Pb(II), blue for Cd(II).

Table 4 shows the identified values of dynamic model parameters, for all experimentally investigated cases. Figure 4 show the variation of ion metal liquid concentration in time, both experimentally obtained and by means of mathematically modelling (Fig. 4a,c), and the sensitivity of mean square deviation of concentration as a function of the effective diffusion coefficient for single silent and US-assisted adsorption (Fig. 4b,d). The results indicate an accurate and valid identification of model parameters. Experimental and model based predicted ion metal concentration dynamic in time for liquid phase, is presented in Fig. 4e,f.

The data from Tables 3 and 4 and the analytical expression of the equilibrium separation efficiency were obtained using Statistica 10 software, with Matchad and Matlab graphical interface.

The above presented allow highlighting several aspects of general and particular interest: i) the dynamic of US-assisted adsorption is almost 30 times faster than the dynamic in classic, silent contacting (Fig. 4a vs. Figure 4c, Fig. 4e vs. Figure 4f); ii) Fig. 4 show very similar concentration profiles so we can assume, that disregarding the contacting approach, the adsorption process can be characterized by the same mathematical model; iii) the diffusion coefficients for Pb (II) and Cd (II) in single sorption are, on average, 20 times higher in US field (6.4 10^–7^ cm^2^/s, respectively 6.1 10^–7^ cm^2^/s) than in US absence (2.9 10^–8^ cm^2^/s, respectively 3.5 10^–8^ cm^2^/s); iv) in competitive systems, the diffusion coefficients of Pb(II) and Cd(II) in HS, disregarding the sorption procedure, are very close, as shown in Table 4; v) the diffusion coefficients of Pb(II) and Cd(II) in competitive single sorption are almost 2.5 times lower than in single competitive systems (Table 4); vi) in US-contacting the effect of species interaction on competitive sorption does not appear to be significant (close values of diffusion coefficients in silent and competitive adsorption); vii) overall, the obtained values of diffusion coefficients for Pb(II) and Cd(II) in HS, a porous vegetal structure, correspond with some other reported^47–50^; viii) besides the diffusion coefficients, the major difference between adsorption in silent and US-assisted approach is also indicated by the obtained constant rates, $k_{a}$; thus, as can be seen in Table 4, for single sorption the ratios *k*_*a_USPb*_/ *k*_*a_silentPb*_ and *k*_*a_USCd*_/ *k*_*a_silentCd*_ are both around 14, while in competitive systems *k*_*a_USPb*_/ *k*_*a_silentPb*_ = 3.3 and *k*_*a_USCd*_/ *k*_*a_silent Cd*_ = 5.44; ix) comparing the values of the adsorption rate constants (Table 4) as well as these ratios of constants, it can be concluded that species competition determines a lower frequency of active sorption sites loading.

The obtained results, confirm that the model of sorption dynamics of Pb(II) and Cd(II) on HS is adequate. It was thus possible to identify the diffusion coefficients of Pb(II) and Cd(II), as well as the values of sorption and desorption rates of these species, in their interaction with the HS porous structure, in the absence or presence of the US field.

## Conclusions

The statistical modelling presented in this paper, following a 2^4^ experimental design, was used to evaluate Pb(II) and Cd(II) biosorption in single and competitive US-assisted and silent systems. The sorption efficiency at equilibrium was evaluated as influence by adsorbent particle diameter, liquid phase pH, solid/liquid ratio, and initial ion metal concentration in the liquid phase. Liquid–solid (HS) equilibrium for single Pb(II) and Cd(II) adsorption was modelled using Langmuir equation. A new procedure was applied to obtain the equilibrium isotherms of Pb(II) and Cd(II) competitive sorption in US-assisted and silent approach.

To study the dynamics of the sorption process, unsteady diffusion with competitive sorption and desorption in solid particles was considered. The model equations were developed accordingly and AP was modelled and optimized. The numerical form of the model, represented as a function having the effective diffusion coefficient and desorption rate constant as arguments, made the identification of the two parameters possible. The identification was based on experimental curves representing the dynamics of the liquid concentration of transferable species, in silent and US assisted sorption. To our knowledge, our study reports for the first time numerical values of effective diffusion coefficients and sorption rate coefficients obtained for Pb(II) and Cd(II) removal in US-assisted field using a porous biosorbent (HS in this specific case).

## Abbreviations

*a*: Langmuir adsorption constant, calculated with Eq. (13) (kg/m^3^)
$c_{i}$: Concentration of species *i* in the liquid within solid particle, kg/m^3^
$c_{l0}$: Initial ion metal concentration in the liquid phase, kg/m^3^
*c*_*li*_: Concentration of species *i* in the liquid phase, kg/m^3^
$c_{le}$: Ion metal concentration in the liquid phase at equilibrium, kg/m^3^
$c_{l\tau }$: Ion metal concentration in the liquid phase at time ${\uptau }$, kg/m^3^
*c*_*li,exp*_: Experimental concentration of specie *i* in the liquid phase, kg/m^3^
$c_{li \, mn}$: Momentary mean concentration of species *i* in liquid phase, kg/m^3^
$c_{liexp \, mn}$: Experimental momentary mean concentration of species *i* in liquid phase kg/m^3^
$c_{se}$: Ion metal adsorbed amount per unit mass of biosorbent, kg/kg
$c_{si}$: Concentration of species *i* in solid phase kg/kg
$c_{si \, mn}$: Momentary mean concentration of species *i* in solid phase kg/kg
*d*_*p*_: Biosorbent particle diameter, m
$D_{ef\left( i \right)}$: Diffusion coefficient (of species *i*), m^2^/s
*e*: Index defining equilibrium, dimensionless
*j*: Current number for the time position of $c_{liexp \, mn}$, dimensionless
*k*_*ai*_: Adsorption rate constant of species *i*, s^-1^
*k*_*di*_: Desorption rate constant of specie *i*, s^-1^
K_e_: Biosorption process equilibrium constant, dimensionless
${\text{m}}_{s}$: Amount of adsorbent, kg
*m*: Number to designate experimental approach ($m = 0$ for silent adsorption and $m = 1$ for US assisted adsorption), dimensionless
*m*_*HS*_: Solid liquid ratio, kg/m^3^
*n*: Number to designate the adsorbed species ( for single Pb(II) adsorption, for single Cd(II) adsorption, refers to Pb(II) adsorption in competitive systems and refers to Cd(II) adsorption in competitive systems, dimensionless
*r*: Current particle radius, m
$R$: Maximum removal efficiency, %
$$R_{\tau }$$: Removal efficiency, %
*R*_*p*_: Particle equivalent radius, m
*S*: Mass transfer area in liquid–solid system, m^2^
Q: Adsorbed amount at equilibrium, kg/kg
$v_{Ri}$: Overall adsorption rate of specie *i*, kg/m^3^/s
$V_{l}$: Experimental liquid phase volume, m^3^
*x*_*i*_: (With i = 1 .. 4)dimensionless value of the process factors (independent variable)
*x*_*ci*_: Dimensionless factors value in the centre of orthogonal plane
$\alpha_{i}$: Constant in Eq. (7) showing the sorption sites coverage by *i* species, dimensionless
*β*: Dimensionless coefficients
*ε*_*P*_: Particle porosity, m^3^/m^3^
*ρ*_*P*_: Particle density, kg/m^3^
*τ*: Time, s
