# Author Correction: Full factorial design and dynamic modelling of silent and ultrasound-assisted lead and cadmium removal by porous biosorbent

**DOI:** 10.1038/s41598-022-12454-4

**Published:** 2022-05-17

**Authors:** S. Bdaiwi Ahmed, T. Dobre, F. Hashim Kamar, A. Mocanu, I. M. Deleanu

**Affiliations:** 1 Department of Chemical and Biochemical Engineering, University Politehnica of Bucharest, Polizu 1‑7, 011061 Bucharest, Romania; 2 Environment and Water Directorate, Ministry of Science and Technology, Baghdad, Iraq; 3 Engineering Technical College, Middle Technical University, Baghdad, Iraq

Correction to: *Scientifc Reports* https://doi.org/10.1038/s41598-022-10792-x, published online 28 April 2022

The original version of this Article contained an error in Affiliation 3, which was incorrectly given as ‘Institute of Technology - Baghdad, Middle Technical University, Baghdad, Iraq’. The correct affiliation is listed below.

Engineering Technical College, Middle Technical University, Baghdad, Iraq

The original Article has been corrected.

